# Thrombosis management in times of COVID-19 epidemy; a Dutch perspective

**DOI:** 10.1186/s12959-020-00220-3

**Published:** 2020-04-20

**Authors:** Hugo ten Cate

**Affiliations:** Thrombosis Expertise Center and department of Internal medicine, Maastricht University Medical Center and CARIM school for cardiovascular diseases, Maastricht University, Maastricht, the Netherlands

The current pandemic of COVID-19 infection [1] has caused major shifts in healthcare organization, towards far more and acute attention for patients suspected from COVID-19 infection at the potential cost of less attention for “regular” care. Obviously, the occurrence of this pandemic does not diminish the burden of other diseases, rather the focus is temporarily shifted towards infectious diseases; while this shift in focus is fully appreciable and also necessary to deal with the huge impact of COVID-19 infections throughout the world, it is important not to forget some essential matters that are part of our regular work.

For those dealing with patients at risk of thrombosis, it is essential that care is taken not to miss the occasional patient with pulmonary embolism (PE); why would this happen? At our hospital and many others, the triage of patients suspected from possible COVID-19 infection get a workup including physical examination, routine lab tests and a “blank” computed tomography (CT) scan of the thorax, to exclude the presence of findings suggestive for viral pneumonia. In practice, subjects with a low suspicion for viral pneumonia, *eg* patients with chest pain but associated dyspnea that could also fit with a diagnosis of angina or PE, will still get a CT scan without contrast agent upon triage, to exclude viral pneumonia; if there is no further workup for the initial complaints (thoracic pain), a diagnosis of PE may be missed. While normally, a Wells rule or similar algorithm will be applied to work up such patients (and ECG recording of course) with a d-dimer test in case of a low suspicion for PE, the threat is that in the present situation this part of the diagnostic pathway is forgotten. Hence, it remains critically important to remain alert for the possibility of alternative diagnoses, including PE [2].

Unfortunately, the d-dimer test is also elevated in those with Covid-19 infection, in particular related to poor prognosis [3] so that an abnormal test result will not discriminate in the acute phase (due to poor specificity, like for other acute diseases, including bacterial pneumonia, acute coronary syndrome and aorta dissecans). Early addition of cardiac ultrasound may theoretically be an option to at least detect significant right ventricle overload, but logistically this may not be easy in the busy triage setting. Even more than before, a clinical suspicion of PE by the GP is essential in order to at least suggest the possibility of PE upon referral. This way the radiology workup could be modified to also include a CT angiography.

Severe viral infection is associated with a coagulopathy, in severe cases this may take the form of disseminated intravascular coagulation (DIC) [4, 5]. Thrombocytopenia is a frequent finding in COVID-19 infection and while the etiology may be multifactorial, the occurrence of DIC is one possible mechanism. Like in any other acute and severe infections, there is a risk of venous thromboembolism (VTE) and for this reason thrombosis prophylaxis in regular doses is pivotal, in principle without need for adjustment of low molecular weight heparin (LMWH) doses in case of thrombocytopenia (although in individual cases this should of course always be balanced against bleeding risk) [2]. Early findings also indicate a possible protective effect of LMWH in those with most severe COVID-19 infections [6].

In our country (the Netherlands), there still is a substantial fraction of patients treated with a vitamin K antagonist (VKA; about 300.000 at the moment), for good reasons (indications like mechanical heart valves), or sometimes less strict reasons (elderly subject with atrial fibrillation and acceptable renal function). Given the requirement of laboratory control (to obtain an international normalized ratio, INR) in all patients on VKA, there is a current tendency among anticoagulation clinics to urge general practitioners to rapidly switch from VKA to a direct oral anticoagulant (DOAC), because of the lack of strict laboratory control, hence avoiding contact between health care worker and patient. While this ad hoc policy makes some sense from the perspective of infection control, it may be potentially harmful if strict requirements for switching are not properly followed. On request of the Dutch general practitioner association (“Nederlands Huisartsen Genootschap”, NHG), dr Geert-Jan Geersing and others provided a concise guidance document that must be followed in case a patient is switched to a DOAC in current emergency setting (in Dutch, [7]). In this brief guidance they highlight the need for switching from VKA to DOAC with care and caution, to choose the right DOAC for the given patient and to carefully consider factors like adherence, renal and liver function, comorbidity and organization of follow up. The general fear being that in haste, such guidance is skipped and patients are being put on a DOAC for simplicity, which may contribute to errors including overlapping periods of anticoagulation, terminating VKA without proper starting DOAC, lack of explanation to the patient of the reasons for such drug changes etcetera; potentially, this introduces a risk of thromboembolism, or bleeding. At the same time, one should consider that routine blood drawing to obtain an INR is always a matter of great care and precaution and in our experience so far, proper arrangements with many patients can be made to reduce the risk of mutual infection in this oftentimes vulnerable population of VKA users. Practically, delaying INR control (and extending dosing regimens) as much as possible in stable patients, or in patients with proven Covid-19 infection, or in those with possible infection of any kind, is often possible without much harm, as long as all such situations are carefully considered by the anticoagulation management team. Good communication among patients and health care workers is critical to ensure mutual safety.

Finally, for all those patients on antithrombotic agents, the risks of bleeding and thrombosis must be weighed even in times of a crisis (certainly with possible drug interactions) and while annual controls of renal function, adherence, assessment of complications and so mostly can be delayed for some time, these checkups should not be abolished altogether, like periodic measurement of blood pressure should not be postponed indefinitely. While in this very intense era of crisis management with its huge impact on all healthcare workers, much attention is correctly directed towards the care for patients with Covid-19 infection, we should find ways to also give attention to the millions of patients at risk of thromboembolism (and bleeding) throughout the world.
